# PolyQ-expanded ataxin-3 protein levels in peripheral blood mononuclear cells correlate with clinical parameters in SCA3: a pilot study

**DOI:** 10.1007/s00415-020-10274-y

**Published:** 2020-10-26

**Authors:** Kathrin Gonsior, Gabriele Anna Kaucher, Patrik Pelz, Dorothea Schumann, Melanie Gansel, Sandra Kuhs, Thomas Klockgether, Sylvie Forlani, Alexandra Durr, Stefan Hauser, Tim W. Rattay, Matthis Synofzik, Holger Hengel, Ludger Schöls, Olaf H. Rieß, Jeannette Hübener-Schmid

**Affiliations:** 1 Institute of Medical Genetics and Applied Genomics, University of Tübingen, Calwerstraße 7, 72076 Tübingen, Germany; 2 Centre for Rare Diseases, University of Tübingen, Tübingen, Germany; 3 DFG NGS Competence Center Tübingen, Tübingen, Germany; 4 Department of Neurology, University of Bonn, Sigmund-Freud-Straße 25, 53127 Bonn, Germany; 5 German Center for Neurodegenerative Diseases (DZNE), Bonn, Germany; 6 Institut du Cerveau-Paris Brain Institute (ICM), Sorbonne Université, AP-HP, INSERM, CNRS, University Hospital Pitié-Salpêtrière, Paris, France; 7 Center for Neurology, and Hertie-Institute for Clinical Brain Research, University of Tübingen, Hoppe-Seyler-Str. 3, 72076 Tübingen, Germany; 8 German Center of Neurodegenerative Diseases (DZNE), Tübingen, Germany

**Keywords:** Ataxin-3, Spinocerebellar ataxia type 3 (SCA3)/Machado–Joseph disease (MJD), Time-resolved fluorescence energy transfer (TR-FRET), Biomarker, Peripheral blood mononuclear cell (PBMC)

## Abstract

In view of upcoming clinical trials, quantitative molecular markers accessible in peripheral blood are of critical importance as prognostic or pharmacodynamic markers in genetic neurodegenerative diseases such as Spinocerebellar Ataxia Type 3 (SCA3), in particular for signaling target engagement. In this pilot study, we focused on the quantification of ataxin-3, the protein altered in SCA3, in human peripheral blood mononuclear cells (PBMCs) acquired from preataxic and ataxic SCA3 mutation carriers as well as healthy controls, as a molecular marker directly related to SCA3 pathophysiology. We established two different highly sensitive TR-FRET-based immunoassays to measure the protein levels of either total full-length, non-expanded and expanded, ataxin-3 or specifically polyQ-expanded ataxin-3. In PBMCs, a clear discrimination between SCA3 mutation carrier and controls were seen measuring polyQ-expanded ataxin-3 protein level. Additionally, polyQ-expanded ataxin-3 protein levels correlated with disease progression and clinical severity as assessed by the Scale for the Assessment and Rating of Ataxia. Total full-length ataxin-3 protein levels were directly influenced by the expression levels of the polyQ-expanded ataxin-3 protein, but were not correlated with clinical parameters. Assessment of ataxin-3 levels in fibroblasts or induced pluripotent stem cells allowed to distinguish mutation carriers from controls, thus providing proof-of-principle validation of our PBMC findings across cell lines. Total full-length or polyQ-expanded ataxin-3 protein was not detectable by TR-FRET assays in other biofluids like plasma or cerebrospinal fluid, indicating the need for ultra-sensitive assays for these biofluids. Standardization studies revealed that tube systems, blood sampling, and PBMC preparation may influence ataxin-3 protein levels indicating a high demand for standardized protocols in biomarker studies. In conclusion, the polyQ-expanded ataxin-3 protein is a promising candidate as a molecular target engagement marker in SCA3 in future clinical trials, determinable even in—easily accessible—peripheral blood biomaterials. These results, however, require validation in a larger cohort and further standardization of modifying conditions.

## Introduction

As for many other neurodegenerative diseases, there is no disease-modifying therapy for Spinocerebellar Ataxia Type 3 (SCA3) today [1]. SCA3 is an autosomal-dominantly inherited neurodegenerative disease which is caused by an expansion of CAG-repeats in exon 10 of the *ataxin-3* (*Atxn3*) gene leading consecutively to a higher number of glutamines (polyQ) in the ataxin-3 protein [2, 3]. The polyQ repeats cause symptoms of ataxia if more than 50 glutamines are expressed [4]. Even though this polyQ-expanded form of the ataxin-3 protein is expressed ubiquitously in somatic cells, selective neurodegeneration of deep cerebellar nuclei and basal ganglia is observed in SCA3 [5, 6]. The physiological form of ataxin-3 protein is located in cell plasma whereas the polyQ-expanded protein is usually found both in cell plasma and nucleus and tends to form intranuclear aggregates in specific neurons during disease progression [7]. Ataxia onset usually occurs at age 30–40 years [1]. The clinical presentation, in addition to gait ataxia, dysarthria, and diplopia [8, 9], is highly diverse and can present as parkinsonian phenotype [10], sleep-associated movement disorder, restless legs syndrome [11], or with psychiatric symptoms as depression [12]. Genetic testing is the gold standard in diagnostics as the disease’s penetrance is 100% [1, 13]. Up to now, the individual progression of disease is monitored clinically by ataxia scales like the Scale for the assessment and rating of ataxia (SARA) [14]. However, today there are no clear molecular biomarkers neither in cerebrospinal fluid (CSF), blood plasma, or serum nor in isolated blood cells like Peripheral Blood Mononuclear Cells (PBMC) to monitor disease onset or progression [15]. As the progression of SCA3 is slow, there is also a significant need for the investigation of easily accessible molecular biomarkers as surrogate endpoints for interventional trials testing new disease-modifying drugs [15]. Therefore, it is crucial that newly established biomarkers are disease specific and that there is a correlation between surrogate and clinical endpoints [16]. As for many other neurodegenerative diseases, neurofilament light chain (NfL) is shown to be increased in preataxic mutation carriers already 7.5 years before disease onset also in SCA3 even correlating with disease severity [17–19]. However, NfL only reflects general axonal damage which captures disease progression and disease severity and, therefore, acts as a progression and severity biomarker. For ataxin-3 the main expected biomarker function would thus be classified as pharmacodynamics/ response biomarker which directly capture target engagement of the key disease protein (FDA classification based upon the context of the use of a biomarker, see https://www.fda.gov/drugs/biomarker-qualification-program/context-use).

In this study, we, therefore, focused on the disease protein—ataxin-3—itself. As multiple targeted disease-protein lowering therapies in polyQ diseases like HD or SCA3 are currently being developed, there is a pressing need for sensitive (molecular) biomarkers for target engagement. For SCA3, it was previously shown that targeting the expanded *ataxin-*3-allele by exon-skipping or *ataxin-*3 gene suppression using antisense oligonucleotides (ASO) in SCA3 mouse models result in lower polyQ-expanded ataxin-3 levels and in a reduction of aggregation [20–22]. Similar results are shown if the down-regulation of the disease protein ataxin-3 via specific micro-RNA (miRNA) is induced [23–26]. To get prepared for ataxin-3 lowering therapies in further clinical trials, our study aimed to establish sensitive methods to measure total full-length and polyQ-expanded ataxin-3 protein in PBMCs, an easily accessible human biomaterial. Additionally, influencing factors as tube systems or the use of different sample processing protocols were validated to adapt highly standardized procedures.

## Methods

### Study subjects

Human PBMC biosamples of 24 ataxic SCA3 patients, four preataxic mutation carriers (i.e. SARA < 3 points), and 31 gender- and age-matched controls were collected at three European centers (Tübingen, Bonn, and Paris). Parallel to blood sampling, the following clinical parameters were acquired: age at onset (AAO) of ataxic symptoms, disease duration (DD), SARA score (0–40 points, 0 points: no ataxia, 40 points: most severe ataxia), and the cross-sectional disease progression (CSDP) (Table 1). The CSDP is calculated by dividing the SARA score by DD in years to evaluate the speed of the disease progression. For classifying fast versus slow progressors, the SCA3 patient cohort was divided by a median split of the CSDP. For classifying different stages of disease severity, the SCA3 patient cohort was separated into mild (≤ twelve points), moderate (13–23 points), and severely affected patients (≥ 24 points) according to the individual SARA scores. All parameters were correlated with the measured protein levels of the polyQ-expanded ataxin-3 and full-length ataxin-3 (which contains polyQ-expanded and physiological ataxin-3) proteins, respectively. Furthermore, control studies with six healthy controls collected under identical conditions were performed to analyze possible influencing parameters of the blood tube sampling systems.

**Table 1:** Subject characteristics

	Ataxic SCA3 subjects	Preataxic SCA3 subjects	Controls*
Sample size (female rate)	24 (10; 41.7%)	4 (1; 25%)	31 (11; 35.5%)
Age in years	52.5 (SD ± 10.3)	30.2 (SD ± 7.6)	44.4 (SD ± 12.6)
Age at onset (AAO) in years	42.4 (SD ± 10.8)	–	–
Disease duration (DD) in years	10.1 (SD ± 6.3)	–	–
SARA score in points	16 (SD ± 8)	0.7 (SD ± 0.6)	–
Cross-sectional disease progression (CSDP^a^) SARA points/years of DD	1.9 (IQR 1.2–2.4)	–	–

*Controls were matched according to sex and age to the ataxic and preataxic SCA3 subjects (± ten years)

^a^CSDP reflects the mean annual disease progression by dividing the SARA score by disease duration. The parameters are reported if they are normally distributed as arithmetic mean and standard deviation (SD) or, if not, as median and interquartile range (IQR)

### Blood/PBMC collection in human subjects

After receiving study consent from all participants, blood collection was performed with one of the following three different blood tube systems: ethylendiamintetraaceat (EDTA), citrate–phosphate–dextrose–adenine (CPDA) tubes (both *S-Monovette*^*®*^*, Sarstedt, DE*) or cell preparation tubes (CPT, *BD Vacutainer*^*®*^* CPT*^*™*^* Mononuclear Cell Preparation Tube, BD Biosciences, Franklin Lakes, US*). To acquire a sufficient amount of PBMC 15 ml whole blood was collected per proband.

### Additional biomaterial from human subjects

Human fibroblasts from a SCA3 patient (female, 70 CAG repeats in the expanded allele) and a sex-matched healthy subject were obtained by skin biopsy and reprogrammed to induced pluripotent stem cells (iPSCs) by electroporation of fibroblasts with episomal plasmids as described by Okita et al. [27]. Generated iPSCs were functionally and genomically validated as described in Hayer et al. [28]. Fibroblasts and iPSCs were analyzed in technical triplicates. Cerebellar human post-mortem brain tissues were received from three male SCA3 patients (mean age 66.8 ± 7.7 years) and three sex- and aged-matched controls (mean age 64.3 ± 16.3 years). Additionally, plasma and CSF samples were obtained from three SCA3 patients (mean age 41 ± 6.5 years, 64–71 CAG repeats in the expanded allele, both gender) and three sex- and aged-matched controls (mean age 43.4 ± 7 years).

### Sample preparation and Bradford

Blood samples from EDTA and CPDA tubes were processed within one hour after blood collection. PBMCs were collected by separation with Ficoll^™^ PM400 (*GE Healthcare Sciences, St. Giles, UK*) after centrifugation at 500 × g for 30 min. Cells were washed in two follow-up steps with Dulbecco’s Phosphate-Buffered Saline (DPBS, *Thermo Fisher Scientific, Waltham, US*). If needed, ery-lysis buffer (NH_4_Cl 155 mM, KHCO_3_ 10 mM, EDTA 250 mM, pH 7.4 0.1 mM) was added to purify PBMCs from red blood contamination. As CPT tubes already contain a separation medium, these samples were directly centrifuged at 1800 × g for 20 min. After centrifugation, cells were snap-frozen at  − 80 °C until further procession. Harvested PBMCs were washed twice with DPBS.

PBMC pellets isolated from all three tubes systems were lysed (lysis buffer: 1 × DPBS, 1% Triton^™^ X-100, 25 × Complete (Protease Inhibitor Cocktail*, Roche Diagnostics, Mannheim, DE*)) for 30 min on ice by vortexing every ten minutes. Lysed PBMCs were diluted with DPBS in the relation of 1:2, and the Bradford protein assay was performed to determine the total protein concentration of each sample [29].

Fibroblasts, iPSCs and post-mortem brain tissue were lysed in RIPA buffer (50 mM Tris, pH 7.5, 150 mM NaCl, 0.1% SDS, 0.5% sodium deoxycholate and 1% Triton^™^ X-100) containing Complete for 25 min on ice, while vortexing every 5 min. Plasma and CSF samples were analyzed without further processing.

### Generation of EGFP ataxin-3

EGFP ataxin-3 plasmids with different polyQ length (15Q, 70Q, and 148Q) were overexpressed in Human Embryonic Kidney (HEK) 293 T cells and used for assay validation and as positive controls during measurements. Per well 600.000 HEK 293 T cells were seeded on a six-well tissue culture plate (*Corning Inc.*) in DMEM medium supplemented with 10% FCS and 1% anti-anti antibiotics-antimycotic (*both Thermo Fisher Scientific*) 24 h before transfection. Transfection followed the Qiagen Transfection Protocol. Shortly, 1.2 µg plasmid DNA, 10 µl OpitMEM (*Thermo Fisher Scientific),* and 4.5 µl Attractene Reagent *(Qiagen)* were mixed and applied with DMEM medium. After an incubation of 48 h, cells were harvested, centrifuged (300 rpm, 5 min), washed with DPBS, and stored at  − 80 °C. Lysis of the cells was performed with 200 µl lysis buffer (1% Triton^™^ X-100, Complete in DPBS) per pellet by incubation on ice for 30 min and vortexing every ten minutes. Protein concentrations were determined using the Bradford protein assay.

### Time-resolved FRET immunoassay

**Fig. 1:**
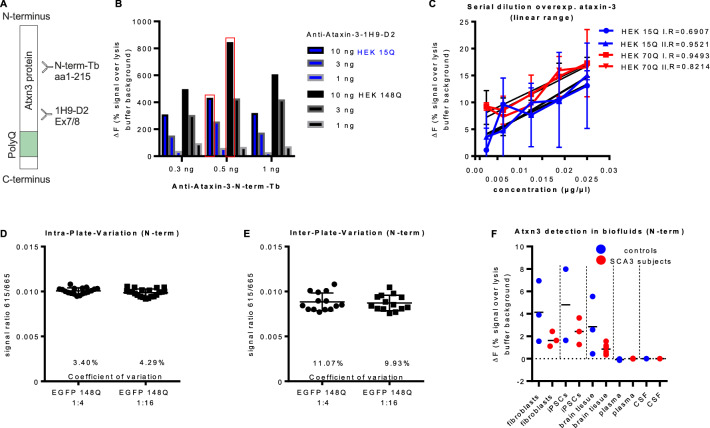
Establishment of a total full-length ataxin-3 TR-FRET-based immunoassay. **a** Schematic representation of antibody binding sites within ataxin-3 protein. Total full-length ataxin-3 is quantified using two ataxin-3-specific antibodies: polyclonal antibody which recognizes the Josephin domain labelled with terbium cryptat (N-term-Tb) and Atxn3 clone 1H9 labelled with D2-fluorophore (1H9-D2). **b** Assessment of the optimal combination of three different concentrations of two specific antibodies N-term-Tb (0.3 ng, 0.5 ng and 1 ng) and 1H9-D2 (1 ng, 3 ng and 10 ng) revealed the highest detected signal using 0.5 ng anti-ataxin-3-N-term-Tb and 10 ng anti-ataxin-3-1H9-D2. **c** A serial dilution with overexpressed ataxin-3 (15Q and 70Q) was performed twice [experiment 1 (I.) and 2 (II.)], detecting a linear range between ataxin-3 protein concentrations of 0.0025–0.025 µg/µl. **d**, **e** Intra- and inter-plate tests showed a coefficient of variation between 3.4 and 4.29% on the same 384-well plate and 11.07–9.93% between samples measured on different 384-well plates. **f** Total full-length ataxin-3 detection in other biofluids than PBMCs failed for plasma and CSF. In homogenates of fibroblasts, iPSCs and brain tissue the detection of total full-length ataxin-3 was partly successful, with a slight distinction between controls and SCA3 subjects

The ataxin-3-specific antibodies ataxin-3 clone 1H9 (*Millipore, MAB5360*) and ataxin-3 N-term (*Abcam, ab96316*) for the detection of the full-length ataxin-3 protein amounts (Fig. 1a), and polyQ antibody clone MW1 (*Study Hybridoma bank*) and ataxin-3 clone 1H9 for the detection of the polyQ-expanded ataxin-3 protein (Fig. 3a) were custom labelled with the fluorophore D2 or Tb, respectively, by *Cisbio Inc*. The labelled antibodies were diluted in detection buffer [50 mM NaH_2_PO_4_, 400 mM NaF, 0.1% BSA, 0.05% Tween20 (*Sigma-Aldrich, St. Louis, US*)]. Per 1 µl detection buffer 10 ng 1H9-D2 and 0.5 ng N-term-Tb, respectively 3 ng MW1-D2 and 0.5 ng 1H9-Tb were added for antibody mixtures. 1 µl antibody mixture and 5 µl of the prediluted PBMC sample was loaded per well (*Proxi-Plate 384 TC plus*), samples were analyzed as duplicate. Time-Resolved Fluorescence Energy Transfer (TR-FRET) measurement was performed with the *Multimode Plate Reader Envision *(*PerkinElmer, Waltham, US*) and the signal ratios between 665 and 615 nm were calculated as raw TR-FRET signals. Afterwards, raw signal was normalized to the measured total protein concentration of each sample and calculated over background signal (deltaF).

### Potential standardization rules for further studies

In our study, we indicated that in further clinical studies highly standardized protocols should be used to minimize center effects. First, all centres should use the same blood collection tubes; for our ongoing validation study, all blood collection for PBMC isolation is done using CPT blood tubes. Importantly for blood collection, if several different blood tubes are collected the order of tubes should be the same for all study probands and additional data like time of last meal (fasting hours) should be reported.

Within 2 h after blood collection, tubes need to centrifuge for 30 min at 1700 rcf at room temperature to reduce red blood contamination. After centrifugation, mononuclear cells and platelets will be visible in the whitish layer under the plasma layer (see the manual of CPT tube system; https://www.bdbiosciences.com/ds/ab/others/PI_CPT_heparin_March_2016_VDP4010507_web_500010323.pdf). After the collection of mononuclear cells using a sterile Pasteur pipette, cells will be washed twice with sterile DPBS and centrifugation at 300 rcf for 15 min at room temperature. Washed PBMCs can be snap frozen at  − 80 degrees and shipped on dry ice for further processing. Importantly, repeated freezing cycles should be avoided by dividing the later protein homogenates into several aliquots.

### Statistical analyses

All statistical and graphical evaluations were performed with *Prism 6* for *Windows*, respectively, *Prism 8* for *Mac* or *Jmp15* for multivariate analyses. To evaluate if a data set was normally distributed (*p* value > 0.05) or not (*p* value  ≤ 0.05) the Shapiro–Wilk test was performed. For normal distributed datasets, group analysis was performed by unpaired *t* test; otherwise, the Mann–Whitney test was used. Normally distributed dependent cohorts were analyzed by paired *t* test and not normally distributed cohorts by Wilcoxon test. Linear correlations for normally distributed data were analyzed with the Pearson correlation coefficient, not normally distributed data with the Spearman correlation coefficient. Linear regression analyses were performed to compare ataxin-3 protein levels to clinical data with adjustment for gender and age. To estimate sample size for the validation cohort, a power of 80%, known median and standard deviation from this pilot study, *p* value of ≤ 0.05 and Cohen’s D effect size were included in the calculation. Statistical significance is demonstrated by *p* values [*p* ≤ 0.05 (*), *p* ≤ 0.01 (**), *p* ≤ 0.001 (***), *p* ≤ 0.0001 (****)].

## Results

### Establishment of the total full-length TR-FRET assay

To establish a TR-FRET-based immunoassay detecting total full-length (physiological and polyQ-expanded) ataxin-3, different antibody concentrations (Tb-labelled anti-ataxin-3-N-term-antibody in 0.3 ng, 0.5 ng and 1 ng and D2-labelled anti-ataxin-3-1H9-antibody with 1 ng, 3 ng and 10 ng) (Fig. 1a) were tested in HEK 293 T cell homogenates overexpressing human ataxin-3 with normal or polyQ-expanded ataxin-3 (15Q, 148Q). The highest deltaF signal occurred in combination with 0.5 ng anti-ataxin-3-N-term-Tb and 10 ng anti-ataxin-3-1H9-D2 for both normal (15Q) and polyQ-expanded (148Q) ataxin-3 (Fig. 1b). Different incubation conditions of several hours by room temperature or by 4 °C revealed the best measurement results after incubation of cell homogenates and antibody mixture for 22 h at 4 °C (data not shown). A serial dilution using overexpressed human ataxin-3 with 15Q or 70Q was performed to examine the linear detection range of the assay. The linear assay range ranked between 0.0025 and 0.025 µg/µl of full-length protein (Fig. 1c). To prove the reliability of the assay, intra- and inter-plate measurements were performed with the known HEK 293 T cell homogenates (148Q). For the intra-plate comparison between measurements, one 384-well plate was loaded randomly with 18 samples of the 148Q homogenate diluted 1:4, and 22 samples diluted 1:16. The analysis showed a coefficient of variation between 3.40 and 4.29% (Fig. 1d). The inter-plate-variation was tested on seven different 384-well plates, measured on different days and loaded with two different sample dilutions (1:4, 1:16 HEK 148Q). The coefficient of variation in the inter-plate measurements was slightly higher (9.93 to 11.07%) compared to the intra-plate test (Fig. 1e). In addition, various other biosamples—including fibroblasts, induced pluripotent stem cells (iPSC), post-mortem brain tissue, plasma, and CSF—were tested as possible biomaterials for ataxin-3 measurements. While in fibroblasts, iPSCs, and post-mortem brain tissue positive deltaF signals were observed, the attempts to detect the full-length ataxin-3 protein in CSF or plasma by this assay failed (Fig. 1f); possibly due to the low levels of ataxin-3 in these biofluids which require ultra-sensitive assay technologies (like, e.g. SIMOA).

### Levels of total full-length ataxin-3 and their correlation to clinical parameters

**Fig. 2:**
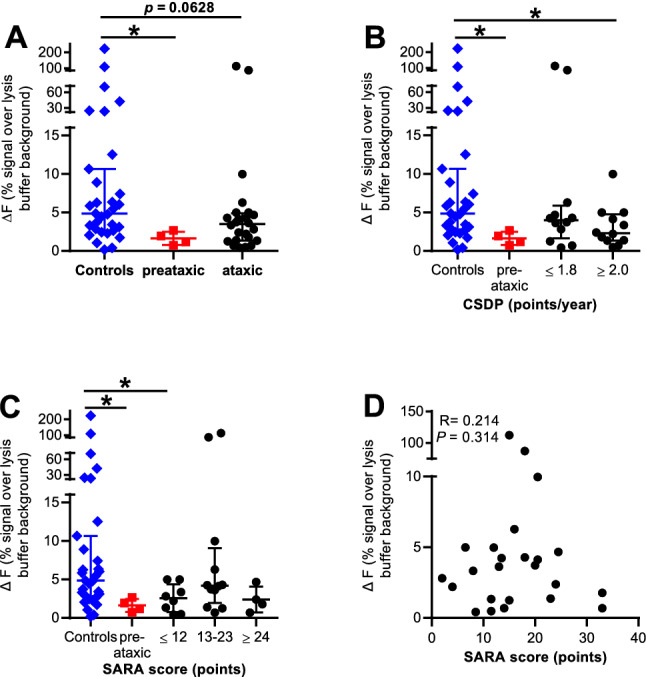
Levels of total full-length ataxin-3 protein and correlation with clinical parameters. The scatter plots visualize the measured ΔF signal of total full-length ataxin-3 protein level in controls, preataxic and ataxic SCA3 subjects. SCA3 patients were divided into subgroups for the disease progression score CSDP and the clinical SARA score to analyze linear correlations. Due to the fact whether there is a normal distribution in one cohort (calculated with Shapiro–Wilk test) central horizontal lines indicate the arithmetic mean or median and the higher and lower bar the range of standard deviation or interquartile range. **a** Significantly less full-length ataxin-3 protein levels in preataxic mutation carriers compared to controls were detected (*p* value 0.0165). Ataxic SCA3 subjects showed lower total full-length ataxin-3 protein levels, which did not reach significance (*p* value 0.0628). Measured values of the control cohort were widely spread. **b** SCA3 patients with a CSDP ≥ 2 points per year (*p* value 0.0259) showed lower total full-length ataxin-3 levels compared to controls. **c** Mildly affected SCA3 patients with a SARA score of ≤ 12 points demonstrated reduced total full-length ataxin-3 protein levels (*p* value 0.0335). **d** Linear correlation analyses revealed no linear correlation of SARA score and total full-length ataxin-3 protein levels (*R* = 0.214; *p* = 0.314; Spearman correlation). Controls are labelled blue, preataxic mutation carriers red and SCA3 patients black

We first compared levels of total full-length ataxin-3 (physiological plus polyQ-expanded) protein between ataxic SCA3 subjects, preataxic SCA3 subjects and controls. In all three groups, total full-length ataxin-3 protein values were widely spread **(**Fig. 2a). Preataxic SCA3 subjects had significantly lower total full-length ataxin-3 protein levels compared to controls (*p* value 0.0165) (Fig. 2a). Ataxic SCA3 subjects also expressed lower total full-length ataxin-3 protein levels, but this did not reach significance (*p* value 0.0628; Fig. 2a). We next correlated total full-length ataxin-3 levels with SARA score and CSDP. Significantly lower total full-length ataxin-3 protein levels were observed for ataxic SCA3 subjects with a fast progression (i.e. CSDP of two or more SARA points per disease year) compared to controls (*p* value 0.0259; Fig. 2b). Ataxic SCA3 subjects with a SARA score lower or equal twelve points (mildly affected) demonstrated significantly lower total full-length ataxin-3 protein levels in comparison to the control cohort (*p* value 0.0335; Fig. 2c). Performing multivariate comparison between the clinical data AAO, disease duration, SARA and CSDP revealed a significant correlation of SARA and disease duration (*p* value 0.0079; *R* = 0.5284) as well as of CSDP and disease duration (*p* value 0.0128; *R* =  − 0.500). Linear correlation analyses demonstrated no linear correlation for each clinical parameter with the total full-length ataxin-3 protein levels (Fig. 2d, and data not shown).

### Validation of polyQ-expanded ataxin-3 TR-FRET-based immunoassay

**Fig. 3:**
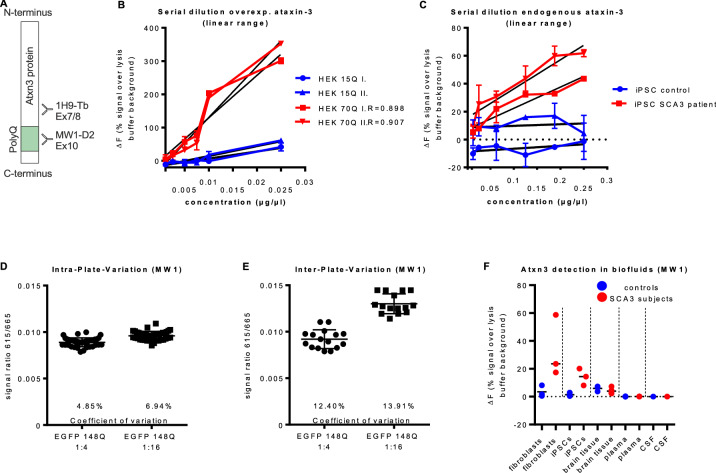
Validation of polyQ-expanded ataxin-3 TR-FRET-based immunoassay. **a** Schematic representation of antibody binding sites within ataxin-3 protein. Expanded ataxin-3 is quantified using the ataxin-3-specific antibody Atxn3 clone 1H9 labelled with terbium cryptat (1H9-Tb) and polyglutamine specific antibody MW1 labelled with D2-fluorophore (MW1-D2). (**B)** A serial dilution using overexpressed ataxin-3 (15Q and 70Q) was performed in two independent experiments (I. and II.) detecting a linear range between concentrations of 0.001–0.025 µg/µl. **c** A serial dilution with endogenous ataxin-3 (control in blue and SCA3 subject in red) gained from iPSCs was performed in two independent experiments. A linear range was detectable between concentrations of 0.01–0.25 µg/µl ataxin-3 protein. **d**, **e** Intra- and inter-plate variations showed a coefficient of variation between 4.85—6.94% for samples measured on the same plate and 12.40–13.91% between samples measured on different plates. **f** Detection of polyQ-expanded ataxin-3 in other biomaterials failed for plasma and CSF. In homogenates of fibroblasts, iPSCs and brain tissue polyQ-expanded ataxin-3 was successful detected and showed mostly discrimination between SCA3 subjects and controls

For the specific detection of polyQ-expanded ataxin-3 protein in PBMC homogenates different antibodies were used: the MW1 D2-labeled antibody binds polyQ-tracts, which are longer than 15Q and was combined with the 1H9 Tb-labelled antibody which recognizes the epitope E214-L233 (Fig. 3a). Assay conditions and antibody concentrations were already established in an earlier study [30] and adapted here to human material. To identify the linear detection range of the assay, different serial dilutions were performed, and each experiment was repeated in a second independent setting. Overexpressed ataxin-3 expressing 15Q or 70Q, respectively, indicated a detection range between concentrations of 0.001 and 1 µg/µl for both analyzed proteins (data not shown). Further validation of the linear range demonstrated a higher specificity of the assay for polyQ-expanded ataxin-3 between 0.001 and 0.025 µg/µl (Fig. 3b). To adapt the assay to endogenous ataxin-3 concentrations, human candidate biomaterials assessing endogenous polyQ-expanded ataxin-3 isolated from iPSCs from SCA3 subjects were used to perform a serial dilution experiment. Here, the linear range ranked between concentrations of 0.01 and 0.25 µg/µl (Fig. 3c). For the intra-plate comparison between measurements, one 384-well plate was loaded randomly with 40 samples of human overexpressed ataxin-3 expressing 148Q isolated from HEK 293 T cells diluted 1:4 and 40 samples diluted 1:16. The coefficient of variation ranked between 4.85 and 6.94% (Fig. 3d). The inter-plate variation was tested on eight 384-well plates, measured on different days, and loaded with two different sample dilutions (1:4 and 1:16 EGFP 148Q). The coefficient of variation in the inter-plate measurements showed values between 12.40 and 13.92% (Fig. 3e). The measurement of polyQ-expanded ataxin-3 with the 1H9-MW1-immunoassay was successful in biomaterials of proteins isolated from fibroblasts, iPSCs, and post-mortem brain tissue, mostly showing a distinction between SCA3 patients and controls. However, as shown before for total full-length ataxin-3, no reliable amounts of polyQ-expanded ataxin-3 were detectable in biofluids where ataxin-3 levels are likely ultra-low, namely plasma and CSF (Fig. 3f).

### Levels of polyQ-expanded ataxin-3 protein and their correlation with clinical parameters

**Fig. 4:**
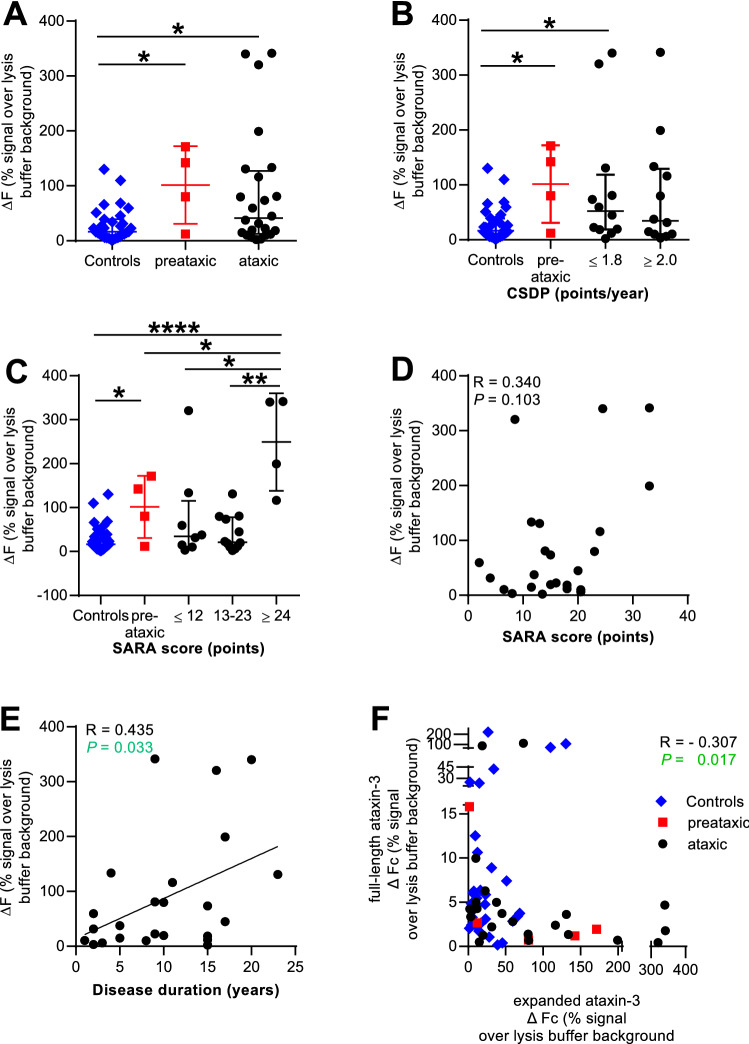
Levels of polyQ-expanded ataxin-3 protein and correlation with clinical parameters. The scatter plots visualize the measured ΔF signal of polyQ-expanded ataxin-3 protein levels in controls, preataxic mutation carrier and ataxic SCA3 patients. SCA3 patients were divided into subgroups for the different clinical parameters. **a** PolyQ-expanded ataxin-3 protein levels discriminate between ataxic (*p* value: 0.0324) and preataxic SCA3 subjects compared to controls (*p *value 0.0372). **b** SCA3 subjects with a CSDP of ≤ 1.8 points per year (*p* value 0.0397) expressed significantly more polyQ-expanded ataxin-3 than controls. **c** The highest ataxin-3 protein values were found in severely affected SCA3 patients with a SARA score ≥ 24 points [*p *value (control): < 0.0001; *p* value (preataxic): 0.0305; *p* value (mild affected, < 12 SARA score points): 0.0283; *p *value (moderate affected, 13–23 SARA score points): 0.002]. **d** Correlation analyses revealed no significant correlation between SARA score and polyQ-expanded ataxin-3 protein level (*p* value 0.103, *R* = 0.340, Spearman’s correlation). **e** PolyQ-expanded ataxin-3 protein level shows a significant positive linear correlation with disease duration (*p* value 0.033; R = 0.435, Spearman correlation). **f** A significant negative linear correlation was detected for total full-length and polyQ-expanded ataxin-3 protein levels (*p* value 0.017, *R* =  − 0.307; Spearman correlation). The graph illustrates measured ΔF signal of polyQ-expanded ataxin-3 protein levels on the abscissa axis and measured ΔF signal of full-length ataxin-3 protein levels on the ordinate axis. Controls are labelled blue, preataxic mutation carriers red and SCA3 patients black

Using the assay of polyQ-expanded ataxin-3, we discriminate both preataxic mutation carriers (*p* value 0.0372, Fig. 4a) and ataxic SCA3 patients from controls (*p* value 0.0324, Fig. 4a). Correlating polyQ-expanded ataxin-3 protein level to disease progression, ataxic SCA3 patients with a slow disease progression (1.8 or fewer SARA points per disease year) express significantly higher levels of the polyQ-expanded ataxin-3 protein as controls (*p* value 0.0397, Fig. 4b). Correlation with SARA revealed that the severely affected SCA3 patients (24 points or more) showed the highest polyQ-expanded ataxin-3 protein levels compared to moderate (13–23 points; *p* value 0.002), to mild affected SCA3 patients (twelve or less points; *p* value: 0.0283) and to preataxic mutation carriers (*p* value 0.0305) (Fig. 4c). Furthermore, in linear regression analysis polyQ-expanded ataxin-3 protein levels showed a tendency for a positive linear correlation with the SARA score (*p* value 0.103; *R* = 0.340; Fig. 4d) and a positive linear correlation with disease duration (*p* value 0.0332; *R* = 0.435; Fig. 4e). A direct comparison between total full-length and polyQ-expanded ataxin-3 revealed that patients with higher levels of polyQ-expanded ataxin-3 protein had lower levels of full-length ataxin-3 protein (*p* value 0.017; *R* =  − 0.307; Fig. 4f).

### Standardization of the procedure and measurement

**Fig. 5:**
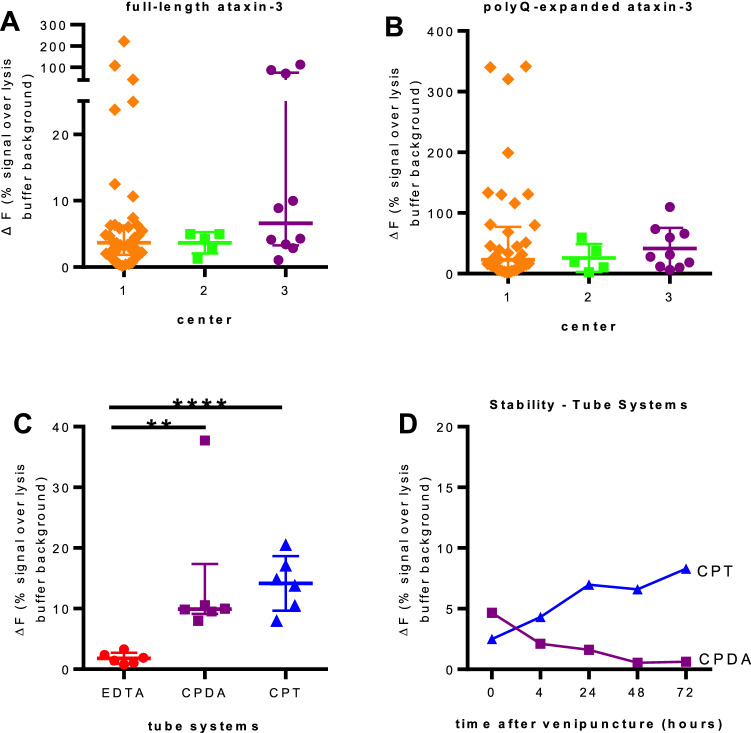
Standardization of the procedure and measurement. Biomaterials for this pilot study were collected at three different European centers under not consistent protocols with different blood tube systems and PBMC isolation protocols. **a**, **b** Total full-length ataxin-3 protein levels are widely spread in biosamples received from center 1 and center 3 and polyQ-expanded ataxin-3 protein level in PBMCs isolated from center 1 (**c**) PBMCs isolated from EDTA, CPDA and CPT blood sampling from 3 male and 3 female healthy controls were compared and revealed the lowest signal of total full-length ataxin-3 in PBMCs isolated from EDTA blood tube systems compared to CPDA tubes (*p* value 0.0022) and CPT tubes (*p* value  < 0.0001). **d** The stability of the samples for several hours after venipuncture was tested with both tube systems: CPDA and CPT. Therefore, several blood tubes from a healthy control were collected and incubated at room temperature for several hours (representing the shipment process). Measurement of total full-length ataxin-3 revealed the highest stability of PBMC isolated from CPDA tubes directly after venipuncture and a decrease up to 0 within 72 h. PBMCs isolated from CPT tube system seem to be stable over time

To ensure the reliability of the ataxin-3 measurement in PBMC samples collected at three different centers, the ataxin-3 protein levels of full-length and polyQ-expanded ataxin-3 were compared between these three collection centers (Fig. 5a, b). Center 3 showed a very high standard variation in total full-length ataxin-3 levels. As all provided PBMC samples were isolated from different tube systems and processed by different in-house standard protocols, we compared the different tube systems to standardize blood sampling and sample processing for further studies. Evaluation of the different tube systems for the blood collection revealed that in CPT tubes the highest deltaF signal was measurable, compared to CPDA or EDTA tube systems (Fig. 5c). Linking this knowledge to the different isolation strategies of PBMCs within the different centers revealed that center 1 and center 3 used EDTA and CPDA tubes for isolation also demonstrated the highest variability in measurements (Fig. 5a). Additionally, the stability of the ataxin-3 protein in different tube systems after the venipuncture was tested by incubating blood within different tube systems for different time points (0, 4, 24, 48, 72 h). Protein homogenates from CPT tube systems showed the best stability with increasing deltaF signal with time, while proteins isolated from CPDA tubes demonstrated the highest protein levels initially which was decreasing with time (Fig. 5d).

## Discussion

As SCA3 is a rare, autosomal-dominantly inherited neurodegenerative disorder with a slow disease progression, there is an urgent need for biomarker establishment to accomplish randomized clinical trials (RCT) [31], in particular of pharmacodynamics/ response biomarker which captures target engagement and is even accessible in peripheral blood. Our study demonstrates for the first time a systematic analysis of total full-length and polyQ-expanded ataxin-3 protein levels across human biomaterials linked to clinical data. Learning from similar analogous TR-FRET approaches to assess polyQ-expanded huntingtin in PBMC in HD [32] we developed two sensitive TR-FRET-based immunoassays to measure soluble total full-length ataxin-3 and specifically polyQ-expanded ataxin-3 in PBMC of SCA3 subjects. Our immunoassays demonstrated highly reliable measurements with less than 5% intra-plate variations.

Measurement of total full-length ataxin-3 protein level in 24 ataxic SCA3 subjects showed no significant change of ataxin-3 total full-length protein levels, compared to control subjects. The significant lower total full-length ataxin-3 protein levels in four preataxic SCA3 subjects compared to controls are potentially an effect of the small preataxic cohort size (*n* = 4). However, also the observation of significantly reduced protein amounts in mildly affected SCA3 subjects strengthens the hypothesis that total full-length protein levels are reduced before and directly after disease onset.

PolyQ-expanded ataxin-3 levels can clearly discriminate ataxic and preataxic SCA3 subjects from healthy controls, indicating their potential to serve as potential target-engagement biomarkers. Correlation analyses of these levels with clinical data revealed significant higher polyQ-expanded ataxin-3 values in severely affected ataxic SCA3 subjects, with longer disease duration and slower disease progression and positive linear correlation with disease duration and SARA-based disease severity. No linear correlation was detected of total full-length ataxin-3 with clinical data.

A direct comparison between total full-length and polyQ-expanded ataxin-3 indicated that patients with higher levels of polyQ-expanded ataxin-3 protein had lower levels of total full-length ataxin-3 protein, thus suggesting a reciprocal relationship between both types of ataxin-3 fragments in SCA3. A hallmark of SCA3 and other polyQ-diseases is an aggregation propensity of polyQ-expanded disease protein in neurons. In PBMCs aggregate formation was not reported for any polyQ disorder. In a previous study, we could show that non-expanded a polyQ-expanded ataxin-3 full-length proteins are cleaved by calpains in neurons and muscle tissue, which indicated a potential cleavage of the ubiquitously expressed full-length ataxin-3 protein in all somatic cells [33]. We described two different calpain cleavage sites within full-length ataxin-3 at amino acid position aa 208 and aa 256 [34]. The ataxin-3 antibody clone 1H9 binds the epitope E214-L233 of full-length ataxin-3 protein and—in combination with the N-terminal ataxin-3 antibody—also N-terminal-derived fragments of calpain cleavage at aa 256. In contrast, the 1H9 antibody in combination with the polyQ-specific antibody MW1 to determine polyQ-expanded ataxin-3 protein also detects C-terminal-derived fragments resulting from calpain cleavage at aa 208 and aa 256. Thus, the reciprocal relation between polyQ-expanded ataxin-3 and physiological ataxin-3 protein levels in PBMCs may not represent an effect of recruitment of physiological ataxin-3 into aggregates—as described for neurons-, but might be explained by the simultaneous detection of full-length and N- or C-terminal-derived ataxin-3 cleavage fragments, respectively.

Due to the small sample size and missing standardization in blood sampling and PBMC isolation as well as usage of different blood tube systems in this study, the results need to be interpreted cautiously. Further studies using a larger cohort and a longitudinal study design are warranted. Sample size estimation for the validation cohort calculated a need of around 170–225 SCA3 mutations carriers using ataxin-3 as a readout for disease severity and disease progression (*p* ≤ 0.05; power 80%, effect size 0.8) using the non-standardized protocols as used in this pilot study. Our standardization experiments demonstrated that the CPT blood tube system seems to represent the best system for further studies, given the higher stability of cells over 72 h compared to CPDA and EDTA blood tube systems. We recommend using the CPT blood tube systems with centrifugation within two hours after blood sampling to reduce the contamination of PBMCs with red blood cells and to allow shipment of PBMCs within around three days between study centers without further processing. Additionally, PBMCs can be snap frozen after collection and an additional washing step with sterile DPBS at  − 80 degrees and shipped on dry ice between centers.

Similar to HD, our immunoassays were not sensitive enough to measure ataxin-3 in low-volume biofluids like serum, plasma or CSF. Conditions of the ataxin-3-specific immunoassays thus need to be adapted to a more sensitive assay platform (like, e.g. SIMOA, SMC^™^), as already done for HD [35, 36]. PBMCs are easy, minimally invasive accessible cells that are often studied in neurodegenerative diseases [32, 37, 38]. In the last years, it was often demonstrated, especially in numerous NfL studies in neurodegenerative diseases, that levels of neuronal-derived proteins in CSF are directly linked to protein amounts in peripheral blood [19, 39]. Therefore, measurements of ataxin-3 in PBMCs or—by more sensitive assays—in serum, plasma or CSF can pave the way for further clinical trials to track the disease progression and therapeutic response longitudinally. Our immunoassays are able to analyze ataxin-3 protein amounts under protein-lowering therapies like ASO or miRNA approaches as demonstrated by a miRNA approach using our SCA3 knock-in mouse model [26], thus representing promising pharmacodynamics/response biomarker which capture target-engagement in easily accessible peripheral blood.

In summary, we were able to establish two different TR-FRET-based immunoassays to measure (I) total (physiological and polyQ-expanded) full-length ataxin-3 and (II) polyQ-expanded ataxin-3 in PBMCs of SCA3 subjects. Both immunoassays demonstrated reliable measurements of ataxin-3 protein levels between 0.001 and 0.025 μg/μl ataxin-3 protein amounts. Our immunoassays were able to measure ataxin-3 protein in PBMCs, fibroblasts, iPSCs, and human post-mortem cerebellar tissue. They thus present promising target engagement/ pharmacodynamic parameters in variable tissues that are readily available for both future preclinical and partly also clinical treatment trials.
